# Characteristics of Cerebral Sinus Venous Thrombosis Patients Presenting with Intracerebral Hemorrhage

**DOI:** 10.3390/jcm11041040

**Published:** 2022-02-17

**Authors:** Naaem Simaan, Jeremy Molad, Shlomi Peretz, Andrei Filioglo, Eitan Auriel, Hen Hallevi, Estelle Seyman, Rani Barnea, José E. Cohen, Ronen R. Leker, Asaf Honig

**Affiliations:** 1 Department of Neurology, Ziv Medical Center, Safed 13100, Israel; naaems@ziv.gov.il; 2 Azrieli Faculty of Medicine, Bar Ilan University, Safed 13115, Israel; 3 Department of Stroke and Neurology, Tel Aviv Sourasky Medical Center, Tel Aviv 69978, Israel; jeremymolad@gmail.com (J.M.); henh@tlvmc.gov.il (H.H.); seyman.estelle@gmail.com (E.S.); 4 Department of Neurology, Rabin Medical Center, Petach Tikva 49100, Israel; shlomipe@clalit.org.il (S.P.); eitanman1@gmail.com (E.A.); ranibarnea@gmail.com (R.B.); 5 Department of Neurology, Hadassah-Hebrew University Medical Center, Jerusalem 91120, Israel; afilioglo@gmail.com (A.F.); leker@hadassah.org.il (R.R.L.); 6 Faculty of Medicine, Tel Aviv University, Tel Aviv 69978, Israel; 7 Department of Neurosurgery, Hadassah-Hebrew University Medical Center, Jerusalem 91120, Israel; jcohenns@yahoo.com

**Keywords:** cerebral venous sinus thrombosis, intracerebral hemorrhage, superior sagittal sinus thrombosis

## Abstract

Patients with cerebral venous sinus thrombosis (CVST) occasionally present with intracerebral hemorrhage (ICH). In this study, we aimed to identify predictors for ICH in CVST patients. Prospective CVST databases from three academic centers were retrospectively analyzed. CVST patients with and without ICH upon presentation were compared. Among the 404 included patients (mean age 41.8 years, 33% male), 74 (18.3%) had an ICH. The patients with ICH were older (45 ± 20.6 vs. 41.1 ± 18 years, *p* = 0.045), and were more often pregnant or postpartum women (15% vs. 6%, *p* = 0.011), or chronically hypertensive (15% vs. 5%, *p* = 0.001). The ICH patients had higher rates of seizures (60% vs. 15%, *p* < 0.001), and focal neurological deficits (53% vs. 23%, *p* < 0.001). The ICH group had lower rates of excellent outcome measured by 90-day mRS 0 (56.7% vs. 80.3%, *p* < 0.001) and higher rates of 90-day mortality (8% vs. 3%, *p* = 0.041). Radiological variables associated with ICH included superior sagittal sinus (SSS) thrombosis (63% vs. 36%), isolated cortical vein thrombosis (38% vs. 8%), and presence of venous infarction (34% vs. 7%) (*p* < 0.001 for all). Upon multivariate analysis, chronic hypertension (OR 3.7, *p* = 0.027), being either pregnant or postpartum (OR 4.3, *p* = 0.006), isolated cortical thrombosis (OR 3.5, *p* = 0.007), and SSS involvement (OR 3.4, *p* < 0.001) were independently associated with ICH upon admission. In conclusion, among CVST patients, the following present higher for ICH: pregnant or postpartum women, and individuals with chronic hypertension, cortical vein, or SSS involvement.

## 1. Introduction

Up to 40% of patients with cerebral venous sinus thrombosis (CVST) present with an intracranial hemorrhage (ICH) [1,2], which presents a challenge to anticoagulation therapy [3]. Moreover, in the International Study on Cerebral Vein and Dural Sinus Thrombosis (ISCVT), the presence of ICH was implicated as a poor prognostic factor [4,5]. Patients presenting with ICH in the study were older and had a more severe clinical presentation with either seizures, coma, or focal neurological deficits. However, the ISCVT was performed over 20 years ago; since then, awareness has increased, and improved diagnostic measures have been introduced. We aimed to update our understanding of the frequency of ICH in CVST patients and develop a profile of their clinical and radiological characteristics.

## 2. Materials and Methods

Patients diagnosed with CVST in three large comprehensive stroke centers between 1/2010 and 12/2020 were included in an ongoing prospective dataset and the data were retrospectively analyzed. We identified all patients with CVST aged 18 and above but excluded from this analysis patients diagnosed with CVST younger than 18, or secondary to head trauma, otolaryngeal, or neurosurgical procedures (*n* = 157). 

All included patients were diagnosed with CVST based on cerebrovascular imaging (CT venography, MR venography, or digital subtraction angiography) that was interpreted by both an experienced neuroradiologist and an experienced stroke neurologist. We recorded the side and location of the occluded venous channels and scored them as: 1 (superior sagittal sinus (SSS)), 2 (cortical vein), 3 (transverse and sigmoid sinus either with or without jugular), 4 (cavernous sinus), and 5 (deep venous system). The deep venous system was defined to include the internal cerebral veins (veins of Galen and Rosenthal). Multiple vein/sinus involvement was defined as involvement of more than one site according to the above vein scores. The presence of ICH was determined from admission noncontrast CT.

Patients underwent a thorough investigation according to similar institutional protocols. Data regarding patient demographics, possible etiologies, comorbidities, and vascular risk factors were recorded. Pregnancy testing was performed in all females of procreative age and the postpartum period was defined as 6 weeks following labor. A rheumatologic questionnaire was completed for all patients, including any history of arthritis, arthralgia, rash, oral or genital ulcers, and uveitis. Further rheumatologic investigation was performed when needed. Pathergy testing and genetic screening for HLA-B27 and B51 were performed whenever Behçet’s disease was suspected. 

In all patients, complete blood count, routine blood biochemistry, and coagulation profiles were performed. Routine laboratory screening for hypercoagulable states included protein C, protein S, antithrombin III, factor V Leiden, homocysteine, antinuclear antibodies, anticardiolipin antibodies, antibodies to beta 2 glycoprotein 1, lupus anticoagulant, and antibodies to double-stranded DNA. In patients with either thrombocytosis of unknown cause with platelet counts above 450,000/μL, or unexplained polycythemia with hemoglobin of >18 mg/dL for males and >16 mg/dL for females, a genetic analysis for JAK2-V617F mutation detection was performed. In a minority of patients, mainly in idiopathic CVST, the JAK2-V617F mutation was tested despite normal blood counts at the discretion of the treating physician. In patients with CVST of unknown etiology, a panel of neoplastic markers was performed, including prostate-specific antigen (PSA), cancer antigen 125 (CA 125), carcinoembryonic antigen (CEA), carbohydrate antigen 19-9 (CA19-9), alpha-fetoprotein (AFP), and human chorionic gonadotropin (HCG). Chest, abdomen, and pelvis CT were performed in patients aged ≥50 years. Patient follow-up, ranging between 3 months and 5 years after the index event, was performed in stroke outpatient clinics of the participating centers. 

In the current analysis, we compared data for CVST patients with and without ICH. 

Local institutional review boards approved the study with an exemption from obtaining individual informed consent forms due to the anonymized nature of data collection and the retrospective analysis of data.

Statistical analysis was performed with SPSS version 25 (IBM, Chicago, IL, USA). The two-sample Student’s t-test was applied for testing differences between the study groups for quantitative parameters. Nonparametric tests were used for the comparison of medians with interquartile ratios. Pearson’s chi-square or Fisher’s exact tests were applied for testing the differences between the groups for the categorical parameters. A *p* value ≤ 0.05 was considered statistically significant. Multivariate logistic regression models controlling for age, gender, venous infarction, and all variables that yielded a *p* value < 0.1 were used to control for possible confounders.

## 3. Results

A total of 404 CVST patients (mean age 41.8 ± 18.7, 67% females) were included in the study. Among them, 74 patients (18.3%) had an ICH. CVST patients with an ICH were older (45 ± 20.6 vs. 41.1 ± 18 years, *p* = 0.045) with an apparent tendency towards being female (74% vs. 65%, *p* = 0.12; Table 1). Pregnancy and the peripartum period were significantly associated with ICH (15% vs. 6%, *p* = 0.011). When pregnant or postpartum females were excluded, there was no association between ICH and gender (*p* = 0.285). No significant differences were found in the ethnic distribution between groups. A higher percentage of CVST patients with ICH suffered from hypertension (15% vs. 5%, *p* = 0.001). The rates of other potential risk factors, including smoking, hyperlipidemia, obesity, diabetes, malignancies, previous thrombotic events, dehydration, and infections did not significantly differ between groups. There were no significant differences in the frequency of hypercoagulopathies, including antiphospholipid antibodies, protein C/S deficiency, factor V deficiency, factor II mutation, PT 20210, MTHFR, JAK2, thrombocytosis, or hyperhomocisteinemia.

Interestingly, Behçet’s disease was not observed in any of the CVST patients with ICH, although it was seen in 5% of the patients without ICH (*p* = 0.04).

The clinical presentation in the ICH group revealed lower rates of headaches (68% vs. 82%, *p* = 0.002), and higher rates of seizures (60% vs. 15%, *p* < 0.001) and focal neurological deficits (53% vs. 23%, *p* < 0.001). The ICH group had lower rates of excellent outcome measured by 90-day mRS 0 (56.7% vs. 80.3%, *p* < 0.001) and higher rates of 90-day mortality (8% vs. 3%, *p* = 0.041). 

The distribution of the involved vein was also compared for the two groups. Cases of cortical vein involvement (28% vs. 8%, *p* < 0.001) and cases of any SSS involvement (63% vs. 36%, *p* < 0.001) were more common among patients in the ICH group. Conversely, the ICH group had fewer cases of transverse (55% vs. 69%, *p* = 0.014) and sigmoid (49% vs. 69%, *p* < 0.001) sinus involvement. The percentage of patients with venous infarction (34% vs. 7%, *p* < 0.001) was also higher in the ICH group. 

The multivariate analysis for predictors of intracerebral hemorrhage among CVST patients (Table 2) revealed the radiological biomarkers of cortical vein involvement (OR 3.46, 95% CI 1.4–8.5, *p* = 0.007) and SSS involvement (OR 3.4, 95% CI 1.6–7.2, *p* < 0.001) to be independently associated with ICH. In addition, suffering from chronic hypertension (OR 3.7, 95% CI 1.2–11.7, *p* = 0.027) or being either pregnant or postpartum (OR 4.3, 95% CI 1.5–12.2, *p* = 0.006) were independently associated with ICH. 

## 4. Discussion

In the current study, ICH was found in 18.3% of the patients with CVST. Pregnancy and postpartum state, SSS involvement, and cortical venous involvement with cortical infarcts were identified as independent risk factors for the occurrence of ICH. ICH in CVST patients was associated with both lower probability of achieving mRS 0 at 90-day follow-up and an almost threefold rate of 90-day mortality.

The frequency of ICH in the current series was somewhat lower compared to previous reports [1,4,6,7]. This difference could be attributed to both higher awareness and improved diagnostic measures such as magnetic resonance venography, which have led to the diagnosis of CVST in subtler presentations or at an earlier stage. This may have led to earlier initiation of anticoagulant treatment that possibly prevented the occurrence of venous infarctions and ICH. 

Our results confirm the findings of previous studies [1,4] showing that CVST patients with ICH were older. A possible explanation for the association of ICH with older age may be the presence of an atrophied venous collateral system among older patients, leading to increased intravenular pressure and venous rupture in patients with CVST. For instance, it has been suggested that a paucity of anastomotic veins between the cortical and the intramedullary deep venous systems results in juxtacortical hemorrhages [8]. Moreover, as is the case with chronic subdural in the elderly, thinner wall cortical venules are more liable to rupture in an older population. Degenerative changes to the venular cerebral system may be enhanced in patients with a history of hypertension, which was found to be an independent predictor of ICH and may partially explain the higher prevalence of ICH in older ages. Chronic hypertension may cause longstanding arteriopathy with Charcot–Bouchard microaneurysms more liable to bleed in a state of increased capillary pressure.

There are conflicting results with respect to the association between female gender and ICH in CVST. In our study and in a large Turkish study [9], female gender was independently associated with ICH; however, other European cohorts have not found a similar association [1,8]. This may be explained by different underlying etiologies, such as different rates of peripartum women in the different cohorts studied. Indeed, when pregnant and peripartum women were excluded in our study, no association was found between gender and ICH. We found that being pregnant or postpartum was independently associated with ICH. We postulate that the impaired autoregulation of cerebral blood flow during this period [10,11] results in higher end-capillary and intravenular pressure, ultimately leading to ICH. Moreover, autoregulation may be further impaired in postpartum females suffering from intracranial hypotension due to spinal anesthesia [12].

In the current analysis, as with previous reports [1,4,8], CVST patients with ICH presented more often with focal neurological deficits and seizures. Focal neurological deficits may be caused by either the ICH itself or by focal edema or infarction associated with ICH in CVST patients, while seizures may be triggered by the toxic hemosiderin deposits on the brain cortex. Unsurprisingly, our experience was consistent with previous studies that showed that CVST patients with ICH were also reported to have a higher mortality rate [4,13]. CVST presenting with ICH poses an extreme therapeutic challenge when associated with severe thrombocytopenia, such as in the case of post-COVID-19-vaccine CVST [14]. 

Finally, corroborating findings from previous studies, ICH was found more often in CVST patients with venous infarction, isolated cortical involvement, and any involvement of SSS thrombosis. This is not surprising as thrombosis in these locations leads to focal congestion at cortical areas of the brain where venous outflow is more limited. In our multivariate analysis, cortical vein thrombosis, and not cortical infarction, was found to be an independent predictor of ICH. We assume that cortical vein thrombosis causes higher rates of both cortical infarction and ICH; therefore, venous infarction is not a predictor of ICH on its own. 

The strength of our study was its large-scale, multicenter design. The limitations of the current study included its retrospective nature and the fact that patient imaging was reviewed at each site and not centrally. In order to mitigate the risk of bias, all ICH cases were pooled, and we did not attempt to address each type of ICH separately. 

## 5. Conclusions

Our findings indicate that ICH complicating CVST occurs more frequently in either pregnant and postpartum women, or people who suffer from chronic hypertension and those with SSS and cortical vein involvement. These characteristics may enable clinicians to identify patients at greater risk of developing an ICH in the CVST setting, prompting a more aggressive therapeutic approach with early and full anticoagulation in order to potentially prevent venous infarction and ICH.

## Figures and Tables

**Table 1:** Comparison of CVST patient characteristics with and without intracerebral hemorrhage.

Characteristics	With ICH (*n* = 74)	Without ICH (*n* = 330)	*p* Value
Age, mean (SD)	45.0 (20.6)	41.1 (18.0)	**0.046**
Sex female (%)	55 (74)	186 (65)	**0.126**
Jewish ethnicity (%)	63 (85)	268 (82)	0.389
Smoking (%)	12 (16)	65 (20)	0.463
Hyperlipidemia (%)	7 (9)	22 (7)	0.387
Hypertension (%)	11 (15)	16 (5)	**0.001**
Obesity (%)	1 (1)	20 (6)	0.101
Diabetes (%)	4 (5)	18 (5)	0.988
Behcet’s disease (%)	0 (0)	18 (5)	**0.041**
IBD (%)	0 (0)	6 (2)	0.238
Malignancy (%)	14 (19)	52 (16)	0.535
Previous thrombotic events (%)	8 (11)	29 (9)	0.619
**Precipitating triggers**			
Dehydration (%)	2 (3)	9 (3)	0.892
Infections (%)	5 (5)	25 (8)	0.788
Pregnancy/labor	11 (15)	20 (6)	**0.011**
OCP	14 (19)	69 (21)	0.667
**Clinical presentation**			
Headache (%)	50 (68)	272 (82)	**0.002**
Any focal neurological deficit (%)	39 (53)	75 (23)	**<0.001**
NIHSS upon admission, mean (SD)	2.0 (3.8)	0.5 (1.9)	**<0.001**
Seizure (%)	42 (57)	47 (15)	**<0.001**
Vomiting (%)	19 (36)	60 (18)	0.165
Papilledema (%)	12 (16)	91 (27)	**0.026**
Favorable outcome (90-day mRS 0-2)	59 (80)	294 (89)	0.098
Excellent outcome (90-day mRS 0)	42 (56.7)	265 (80.3)	**<0.001**
90-day mortality (%)	6 (8)	11 (3)	**0.041**
**Hematological workup**			
APLA (%)	9 (12)	46 (14)	0.737
Protein C/S deficiency (%)	1 (1)	9 (3)	0.760
Factor V deficiency (%)	6 (8)	24 (7)	0.808
Factor II mutation (%)	2 (3)	7 (2)	0.736
PT 20210 (%)	5 (7)	13 (4)	0.266
MTHFR (%)	5 (5)	17 (5)	0.603
JAK2 (%)	8 (11)	19 (6)	0.123
Thrombocytosis (%)	4 (5)	15 (5)	0.702
Hyperhomocisteinemia (%)	1 (1)	6 (2)	0.796
**Radiological findings**			
Cortical venous thrombosis (%)	21 (28)	28 (8)	**<0.001**
Venous infarction (%)	25 (34)	24 (7)	**<0.001**
Deep (%)	6 (8)	17 (5)	0.241
SSS involvement	43 (63)	114 (36)	**<0.001**
Transverse sinus involvement (%)	41 (55)	228 (69)	**0.014**
Sigmoid sinus involvement (%)	36 (49)	227(69)	**<0.001**
Cavernous sinus involvement (%)	2 (3)	8 (2)	0.9
Multiple veins (%)	26 (35)	83 (26)	0.084

Values represent number of patients unless otherwise stated. IBD = inflammatory bowel disease, OCP = oral contraceptives, APLA = antiphospholipid antibody syndrome, SSS = superior sagittal sinus, NIHSS = National Institutes of Health stroke scale, PT = prothrombin, MTHFR = methylenetetrahydrofolate reductase, JAK2 = Janus kinase 2. Bold numbers signify statistically significant *p* values.

**Table 2:** Multivariate analysis for predictors of intracerebral hemorrhage.

Characteristic	OR	95% CI		*p* Value
Age	1.019	0.997	1.041	0.099
Female sex	2.14	0.091	5.05	0.08
Pregnancy/Labor	4.28	1.507	12.166	**0.006**
Obesity	0.22	0.027	1.776	0.156
Hypertension	3.69	1.165	11.727	**0.027**
Cortical vein involvement	3.46	1.415	8.483	**0.007**
SSS involvement	3.425	1.635	7.178	**0.001**
Venous infarction	1.603	0.715	3.594	0.252

Bold numbers signify statistically significant *p* values.

## Data Availability

The data presented in this study are available on request from the corresponding author. The data are not publicly available due to the requirements of the institutional review boards.
